# Association between *FMR1* mRNA levels and clinical phenotypes in Fragile X syndrome

**DOI:** 10.21203/rs.3.rs-11053577/v1

**Published:** 2026-09-29

**Authors:** Braeden S. Heald, Walker S McKinney, Lauren Schmitt, Kelli C. Dominick, Richard K. Lacher, Sebastian Piloto, Christina Gross, Craig A. Erickson

**Affiliations:** 1 Divisions of Child and Adolescent Psychiatry Cincinnati Children’s Hospital Medical Center, University of Cincinnati College of Medicine, Cincinnati, OH, USA; 2 Children’s Mercy Kansas and University of Kansas Medical Center, University of Cincinnati College of Medicine, Cincinnati, OH, USA; 3 Phelan McDermid Syndrome Foundation, University of Cincinnati College of Medicine, Cincinnati, OH, USA; 4 Department of Psychiatry and Behavioral Neuroscience, University of Cincinnati College of Medicine, University of Cincinnati College of Medicine, Cincinnati, OH, USA; 5 University of Michigan, University of Cincinnati College of Medicine, Cincinnati, OH, USA; 6 Division of Neurology Cincinnati Children’s Hospital Medical Center and the Department of Pediatrics, University of Cincinnati College of Medicine, Cincinnati, OH, USA

**Keywords:** Fragile X syndrome, Molecular marker, Intellectual disability, Adaptive behavior, Phenotypic severity, Gene expression

## Abstract

**Background:**

Fragile X syndrome (FXS) is caused by a CGG repeat expansion in the *FMR1* gene on chromosome X that leads to hypermethylation and reduces or completely silences the expression of *FMR1* mRNA and its protein product, Fragile X Messenger Ribonucleoprotein (FMRP). Since the clinical manifestations of FXS can be variable, there is interest in identifying molecular markers that reflect phenotypic severity. The extent of CGG repeat expansion and resulting hypermethylation varies substantially between patients, suggesting that *FMR1* gene silencing differs between individuals with FXS and could contribute to the phenotypic variability. Previous studies have identified *FMR1* mRNA as a highly reproducible, sensitive and quantitative measure of gene activity. This study builds on these previous studies and assesses the relationship between *FMR1* mRNA and clinical outcomes in FXS.

**Methods:**

We recruited 300 study participants, including males and females across the following diagnostic groups: FXS, fragile X premutation carriers (PMCs), typically developing controls (TDC), and (non-FXS) developmental disability controls (DD). Peripheral blood samples were used for quantifying both *FMR1* mRNA and FMRP (from a Luminex-based assay). IQ and adaptive behavior were evaluated with the Stanford-Binet 5 and Vineland-3 tests, respectively. Analyses examined group- and sex-level associations between *FMR1* mRNA and FMRP, IQ, adaptive behavior, and age across diagnostic groups.

**Results:**

*FMR1* mRNA was substantially reduced in participants with FXS, particularly males, consistent with expected gene silencing. Within the FXS group, higher *FMR1* mRNA levels were associated with higher IQ and better adaptive behavior. These associations were not observed in the TDC or DD comparison groups, suggesting that the relationship is specific to FXS pathology rather than developmental impairment more broadly. *FMR1* mRNA also correlated positively with FMRP in FXS, supporting its validity as a molecular marker. Age-related increases in *FMR1* mRNA were also observed in females with FXS.

**Conclusions:**

Our findings that *FMR1* mRNA correlates with both FMRP levels and clinical outcomes in participants with FXS support its utility as a clinically meaningful molecular marker of phenotypic severity. As an accessible surrogate of disease severity, *FMR1* mRNA may help improve prognostic assessment and support more individualized treatment planning in FXS.

## Background.

The Fragile X Messenger Ribonucleoprotein 1 (*FMR1*) gene encodes Fragile X Messenger Ribonucleoprotein (FMRP), a critical protein involved in synapse formation and the regulation of synaptic plasticity [1, 2]. Fragile X syndrome (FXS), the most common inherited form of intellectual disability and monogenic cause of autism spectrum disorder (ASD), results from a CGG trinucleotide repeat expansion in the 5’ untranslated region of the *FMR1* gene on the X chromosome. Expansions with over 200 CGG repeats (full mutation, FM) typically lead to hypermethylation of the gene’s promoter and CGG region and thus silencing of gene expression, resulting in absent or significantly reduced FMRP [3]. The absence of FMRP is thought to underlie many of the neurodevelopmental and behavioral characteristics of full mutation FXS (FM-FXS), including intellectual disability, anxiety, hyperactivity, and features associated with ASD.

While FM-FXS often results in hypermethylation and silencing of the *FMR1* gene, some individuals with FM alleles exhibit partial methylation or mosaic patterns, resulting in low but detectable levels of *FMR1* mRNA and FMRP [4–6]. Even in some males with hypermethylation and repeat sizes over 200, trace amounts of FMRP are detectable in peripheral blood [4, 5]. Notably, pilot studies show that IQ is positively correlated with FMRP levels in individuals with FXS, suggesting that blood FMRP could serve as a molecular marker informing disease severity [4]. We recently developed a highly sensitive and reproducible Luminex-based assay to quantify FMRP in patient blood; however, the assay is relatively labor-intensive (~2.5 days) and limited in sensitivity due to inherent characteristics of the antibody-based technology. Since *FMR1* mRNA is the direct product of the gene’s activity, *FMR1* mRNA levels can serve as an indicator of the gene’s activity and regulatory state and a surrogate for FMRP levels. Quantitative real-time PCR assays to detect mRNA are faster and more sensitive compared with Luminex-based assays to detect protein. Moreover, if combined with digital droplet PCR technique, this assay can be used to detect absolute mRNA up to the single molecule level [7]. Our group recently developed a sensitive and reproducible quantitative real-time PCR (qRT-PCR) assay to reproducibly determine *FMR1* mRNA levels in peripheral blood, including trace amounts of *FMR1* mRNA in individuals with FXS [5]. Pilot studies showed that these trace amounts of *FMR1* mRNA detected in individuals with FXS are positively correlated with FMRP, suggesting that they are biologically meaningful [5]. The assay also confirmed that Fragile X pre-mutation carriers (PMCs), characterized by 55–200 CGG repeats, have increased *FMR1* mRNA expression as previously described [5, 8, 9].

The phenotypic spectrum of FXS is vast. Research suggests similar factors drive individual differences in IQ among individuals with FXS as in the typically developing population, including environmental factors, social determinants of health, and genetic modifiers [10, 11]. However, these factors only partially explain phenotypic differences, and it is highly likely that genetic or epigenetic differences affecting *FMR1* transcription and translation also contribute to this variability. Therefore, *FMR1* mRNA levels in peripheral blood as a measurement for *FMR1* gene activity and surrogate for FMRP levels could serve as a quantitative and objective readout to aid in diagnosis and development of treatment strategies in FXS.

Indeed, several studies including our own, suggest that *FMR1* gene expression, as measured by *FMR1* mRNA and/or FMRP levels in blood, is correlated with clinical phenotypes in FXS, such as IQ and behavior [4–6]; however, previous studies evaluating the use of blood *FMR1* mRNA levels to predict disease severity were small in size. Large studies examining associations between *FMR1* gene expression, IQ, and behavior in phenotypically comparable individuals with developmental delay carrying normal ranges of CGG repeat numbers in *FMR1* are scarce.

This study aims to expand these previous studies and assess the relation between *FMR1* mRNA expression and clinical outcomes, including IQ and adaptive behavioral functioning, across a large cohort of individuals with FXS, PMCs, typically developing controls (TDC), and individuals with other developmental disabilities (DD) [12, 13]. The overall goal was to assess if *FMR1* mRNA levels are positively correlated with cognitive and adaptive function across these groups and if they are stable across age to evaluate if peripheral *FMR1* mRNA could serve as a potential molecular marker of phenotype severity across development.

## Methods.

### Participants

All study procedures were approved by the Cincinnati Children’s Hospital Medical Center Institutional Review Board (protocol 2013–7327). Participants included individuals with FXS (N = 131), PMCs (N = 52), typically developing controls (TDCs; N = 57), and individuals with other developmental disabilities (DDs; N = 60). All FXS and PMCs were established patients and recruited through the Cincinnati Fragile X Research and Treatment Center. TDCs and individuals with other developmental disabilities were recruited from the community via flyers, hospital referrals, and word-of-mouth. A total of 300 participants with available *FMR1* mRNA measurements were included. Participants with both mRNA measures and each clinical measure are as follows: FMRP (N = 275), Stanford-Binet, Fifth Edition (SB-5; N = 169), and Vineland Adaptive Behavior Scales, Third Edition (Vineland-3; N = 90). See Tables 1–4 for demographic and diagnostic information for these groups.

Clinical data (FMRP, SB-5, Vineland-3) was included if it was obtained within a year of the date of mRNA blood collection. mRNA and other data were collected on the same day for nearly all participants.

### Blood collection and processing

For the *FMR1* mRNA measurements, blood was collected, stored, and processed using our established quantitative real-time PCR (qRT-PCR) assay, as previously described in Straub et al. [5]. FMRP sample collection and processing followed our Luminex-based quantification protocol previously described in Boggs et al. [4].

### *FMR1* mRNA and FMRP assays

The *FMR1* mRNA assay was completed as outlined in Straub et al. [5] with the following modification: to confirm that a qRT-PCR was run successfully, a healthy control (HC) with a well-established *FMR1* mRNA level was included in every plate. The range of this HC final mRNA was adjusted from the original criteria to 5.67E-09 – 1.21E-08 picomolar (pM) as acceptable range for an assay. We made this adjustment based on internal assessments of changes in the HC base levels of *FMR1* mRNA concentration (most likely due to different time points (years apart)) when HC blood was collected for the present and the previous studies.

Workflow of RNA samples, from whole blood collection to cDNA synthesis is as follows: collection of whole blood in a PAXgene RNA tube, stored in accordance with Qiagen recommendations at −80 °C within 30 minutes of collection, processed from thawed whole blood with PreAnalytiX Blood RNA Kit v2, concentration and purity (260/280) of processed samples analyzed on Cytation 3 spectrophotometer immediately after processing, then stored at −80 °C. cDNA synthesis was usually performed later.

RNA samples processed with the PreAnalytiX Blood RNA Kit v2 are stable for several years according to the manufacturer. However, we observed some minor degradation after one year of storage. Thus, concentration and purity of RNA samples processed more than a year after collection were reassessed and adjustments were made for cDNA synthesis as necessary. To avoid multiple freeze-thaws, RNA was aliquoted into 10 μL aliquots.

FMRP measurements were obtained for a subset of participants (N = 275) using blood and sample processing techniques previously described in Boggs et al. [4].

### Stanford-Binet 5^th^ Edition

A subset of participants (N = 169) completed the Stanford-Binet, Fifth Edition (SB-5), a clinician-administered measure of cognitive functioning (i.e., IQ). Deviation scores were calculated using previously reported methods validated for use in FXS to minimize floor effects common in this population [14]. To minimize missing data, we analyzed deviation scores for Abbreviated IQ (ABIQ), calculated from the two routing subtests of the SB-5 (Nonverbal Fluid Reasoning, Verbal Knowledge).

### Vineland Adaptive Behavior Scales, Third Edition (Vineland-3)

A subset of participants (N = 116; 90 FXS, 9 TDC, 17 DD, only results from FXS reported) completed the Vineland Adaptive Behavior Scales, Third Edition, Comprehensive Interview Form (Vineland-3) [15], a measure of adaptive functioning (i.e., adaptive use of skills to function independently across home, school, and the community). We analyzed standard scores from the Adaptive Behavior Composite (ABC) for the Vineland-3.

### Statistical analyses

All statistical analyses were completed in GraphPad Prism 10. Group differences in mRNA expression across the four diagnostic groups were evaluated using 2-way ANOVA tests, followed by Tukey’s multiple comparison posthoc tests to examine pairwise differences between diagnostic groups (e.g., FXS males vs. PMC males) or between sexes within a group (e.g., FXS males vs. FXS females). To control for multiple comparisons, a false discovery rate (Benjamini-Hochberg FDR) procedure was used. Linear associations were assessed between *FMR1* mRNA levels and (separately) FMRP, SB-5 IQ, the Vineland-3 ABC, and age using Pearson correlations. Depending on sample sizes, we report overall correlations for each diagnostic group as well as separated by sex. Due to limited sample sizes and data availability, Vineland-3 ABC is only reported for the FXS group, and no associations between *FMR1* mRNA and cognitive or adaptive behavior measures were evaluated in the PMC group. Statistical significance for all analyses was set at *p* < 0.05 (two-tailed). All data generated in this study are reported in Tables 1–4.

## Results.

### Participant selection:

mRNA data was collected from individuals in four diagnostic groups: fragile X syndrome (FXS), fragile X premutation carriers (PMCs), typically developing controls (TDC), and developmental disability controls (DD, genetically confirmed to not have FXS) (see “Methods” section for details). A total of 300 (179 male and 121 female) unique participant samples that passed the assay quality control criteria set forth in Straub et al. [5] were collected. Demographic information is shown in Table 1 for the complete patient sample, Table 2 for the sub-sample with available FMRP data, and Table 3 for the sub-sample with available SB-5 data. Only participants with FXS are included in Table 4 for the sub-sample with available Vineland-3 data.

### *FMR1* mRNA expression is significantly reduced in males with FXS compared with all other genotypes regardless of sex:

First, we compared *FMR1* mRNA levels in male and female individuals across the four diagnostic groups: FXS, PMC, TDC, and DD (Fig. 1). We detected a main effect of diagnosis (p < 0.0001) and a significant interaction of sex and diagnosis (p = 0.001) but no main effect of sex. Post hoc comparisons showed that males with FXS exhibited significantly lower *FMR1* mRNA levels than all other male groups (p ≤ 0.0001). Similarly, females with FXS had significantly lower *FMR1* mRNA levels compared to female PMCs (**** p < 0.0001) and DD controls (* p < 0.05) but not female TDCs. Notably, *FMR1* mRNA levels within PMC, TDC, and DD diagnostic groups did not significantly differ between males and females. The FXS group was the only diagnostic category to display significant within-group sex differences, with males showing markedly lower *FMR1* expression than females.

### *FMR1* mRNA levels are correlated with higher IQ and greater adaptive behavior in individuals with FXS but not TDC or DD diagnostic groups

Next, we examined associations between *FMR1* mRNA levels and IQ or adaptive behavior in FXS, TDC and DD diagnostic groups (Fig. 2). In individuals with FXS, *FMR1* mRNA levels were positively correlated with IQ scores in both male and female individuals (Pearson’s correlation, males: r = 0.36, *p = 0.0038, females; r = 0.45, *p = 0.0119, and combined: r = 0.53, *p < 0.0001) (Fig. 2A). Likewise, *FMR1* mRNA levels were positively associated with adaptive behavior scores in individuals with FXS (r = 0.32, * p = 0.0022); however, no significant associations were observed when stratified by sex (Fig. 2B). Notably, no significant correlations between *FMR1* mRNA levels and IQ were detected in TDC or DD diagnostic groups (Fig. 2C,D). Correlations between *FMR1* mRNA levels and IQ or adaptive behavior were not assessed in the PMC group due to small sample size. These results suggest that variation in FMRP levels may contribute to variability in IQ and adaptive behavior in FXS but not in the typically developing individuals or in individuals with developmental disabilities.

### *FMR1* mRNA levels and FMRP protein levels are positively correlated in individuals with FXS

To evaluate if *FMR1* mRNA can serve as a surrogate for FMRP expression, we next evaluated if *FMR1* mRNA is positively correlated with FMRP levels. Our analyses showed that *FMR1* mRNA is positively correlated with FMRP in individuals with FXS (n=124, r=0.57, p<0.0001), which was driven by male individuals (male: n=87, r=0.63, p<0.0001; female: n=37, r=0.25, p=0.13) (Fig. 3A). By contrast, FMRP was not significantly correlated with *FMR1* mRNA levels in PMC, DD and TDC groups but showed a non-significant negative trend, potentially suggesting a negative feedback mechanism of mRNA levels on mRNA translation in these groups (PMC: n=43, r=−0.26, p=0.094; DD: n=57, r=−0.07, p=0.61; TDC: n=51, r=−0.22, p=0.13; not split up by sex due to small sample size) (Fig. 3B–D). These results suggest that in individuals with FXS, but not other diagnostic groups, *FMR1* mRNA is a valid proxy readout for FMRP levels.

### *FMR1* mRNA is positively correlated with age in FXS but not in other diagnostic groups

To assess if *FMR1* mRNA levels could serve as molecular marker across the lifespan in FXS, we analyzed if they change with age. A cross-sectional analysis showed that *FMR1* mRNA levels were positively correlated with age in individuals with FXS, i.e., higher in older adults compared with younger adults (n=131, r = 0.28, p = 0.0011, Fig. 4A). Notably, this correlation was driven by females in the group (female: n=41, r = 0.42, p = 0.006; male: n=90, r = 0.079, p = 0.46). There was no correlation between *FMR1* mRNA levels and age in other diagnostic groups (PMC: n = 52, r = −0.01, p = 0.95; DD: n = 60, r = −0.007, p = 0.96; TDC: n = 51, r = 0.18, p = 0.21, Fig. 4B–D).

## Discussion

FXS is phenotypically heterogeneous: some patients are nonverbal, while others are able to attend college or drive a car [16]. It is currently difficult to predict disease severity, which hinders the development of personalized treatment plans. A subset of patients with FXS express trace amounts of *FMR1* mRNA in peripheral blood that are associated with higher IQ, suggesting *FMR1* mRNA could serve as a marker for disease severity [5, 17]. Our findings of a positive association between *FMR1* mRNA and FMRP in males with FXS supports the hypothesis that *FMR1* mRNA levels in peripheral blood of males with FXS can serve as a surrogate for FMRP levels and a molecular marker associated with IQ and adaptive behavior. Moreover, our results suggest that *FMR1* mRNA levels may increase with age in females with FXS.

### Sex differences in *FMR1* mRNA in patients with FXS

We found that *FMR1* mRNA levels are lowest in patients with FXS. This confirms *FMR1* mRNA levels serve as a readout for *FMR1* transcription activity and thus the underlying mutation. Consistent with this, we further demonstrate that *FMR1* mRNA levels are lower in males than in females with FXS. This is most likely due to random X inactivation in females, through which *FMR1* mRNA is either transcribed from the mutated or the healthy allele. Although the extent to which *FMR1* mRNA levels in peripheral blood reflect levels in the brain is not established, our finding that IQ is positively correlated with *FMR1* mRNA in both male and female individuals with FXS further supports the increasing literature suggesting the potential value of peripheral blood *FMR1* mRNA or FMRP as a proxy for its expression in the brain [5, 6, 18, 19]. Future studies of *FMR1* mRNA in, for example, cerebrospinal fluid could provide firmer confirmation that *FMR1* mRNA in peripheral blood is a good surrogate for *FMR1* gene transcription in the brain.

### Associations between *FMR1* mRNA levels and clinical outcomes in FXS

We also found that *FMR1* mRNA is positively correlated with IQ and adaptive behavior in individuals with FXS, but not in premutation carriers, typically developing individuals, or individuals with other developmental delays. This suggests that *FMR1* expression may contribute more strongly to brain function in the context of low baseline expression as is present in individuals with CGG repeat expansions over 200. This is consistent with past studies demonstrating a pattern of “diminishing returns” when examining associations between FMRP and IQ (e.g., no increases in IQ past ~70% of “normal” FMRP expression [13]). Our recent studies illustrated that IQ in FXS follows a normal distribution, and that individual phenotypic variation is only partially explained by other factors such as social determinants of health [10, 11]. The current study further confirms that *FMR1* gene expression is not a limiting factor for brain function beyond FXS.

### Associations between FMRP and *FMR1* mRNA

Our finding that FMRP protein levels are positively correlated with *FMR1* mRNA levels suggest that *FMR1* transcription is the main determinant of FMRP levels and thus function in FXS and that other regulatory factors controlling *FMR1* mRNA translation play a less prominent role in regulating FMRP levels in the context of low *FMR1* mRNA levels in FXS. By contrast, the trend towards a negative correlation of FMRP and *FMR1* mRNA levels in PMC, TDC and DD groups suggests that translational regulation (e.g., through mRNA binding proteins or noncoding RNAs such as microRNAs) may play a more important role in determining FMRP levels and thus function outside of FXS. This finding has important implications for the development of gene replacement in FXS because it suggests that mechanisms downregulating *FMR1* translation should be restored as well to avoid FMRP overproduction.

### Increased *FMR1* mRNA with age in FXS

Our results suggest that *FMR1* mRNA levels may increase with age in FXS, although this is within the context of our cross-sectional study and this should be confirmed using longitudinal designs. *FMR1* mRNA levels may be increased in older participants with FXS due to sampling or selection biases: our participant sample likely reflects higher functioning patients able to regularly visit our FXS clinic and participate in clinical studies, which could skew *FMR1* mRNA levels towards higher levels in older participants. Longitudinal studies assessing *FMR1* mRNA levels over time will be critical to better understand how age affects *FMR1* expression in FXS.

### Strengths and Limitations

A strength of this study is the inclusion of a developmentally delayed (DD) group without FXS diagnosis as an additional control. However, developmental delays in this group are likely etiologically heterogeneous, and these pathogenic variants could also affect *FMR1* [5] through methods and pathways not yet well understood. While we were able to detect differences between the FXS and the DD groups, it might be useful to further stratify the DD diagnostic group based on underlying gene defects. Likewise, future studies are needed to better understand the molecular underpinnings of the differences in *FMR1* mRNA expression in the FXS group. Studies using long-read sequencing to better characterize the CGG repeat expansion may help provide insight into these mechanisms [20].

Second, the qRT-PCR assay is sensitive and reproducible but relies on relative quantification using a standard curve based on recombinant *FMR1* DNA. Future optimization of this assay using, for example, digital droplet PCR to enable absolute quantification and to increase sensitivity could help further develop *FMR1* mRNA quantification into a prognostic tool in FXS.

Last, our study is limited by small sample sizes and variability in external factors such as age and time of the day of blood draw. Larger studies adequately powered to overcome this heterogeneity are needed to further evaluate the value of *FMR1* mRNA expression as a molecular marker of FXS disease severity.

### Conclusion

This study shows that *FMR1* mRNA levels in peripheral blood of individuals with FXS are positively correlated with FMRP levels, IQ and adaptive behavior. These results suggest blood *FMR1* mRNA levels could be used to predict disease severity and could constitute a first step towards individualized treatment strategies in FXS.

## Figures and Tables

**Figure 1.**
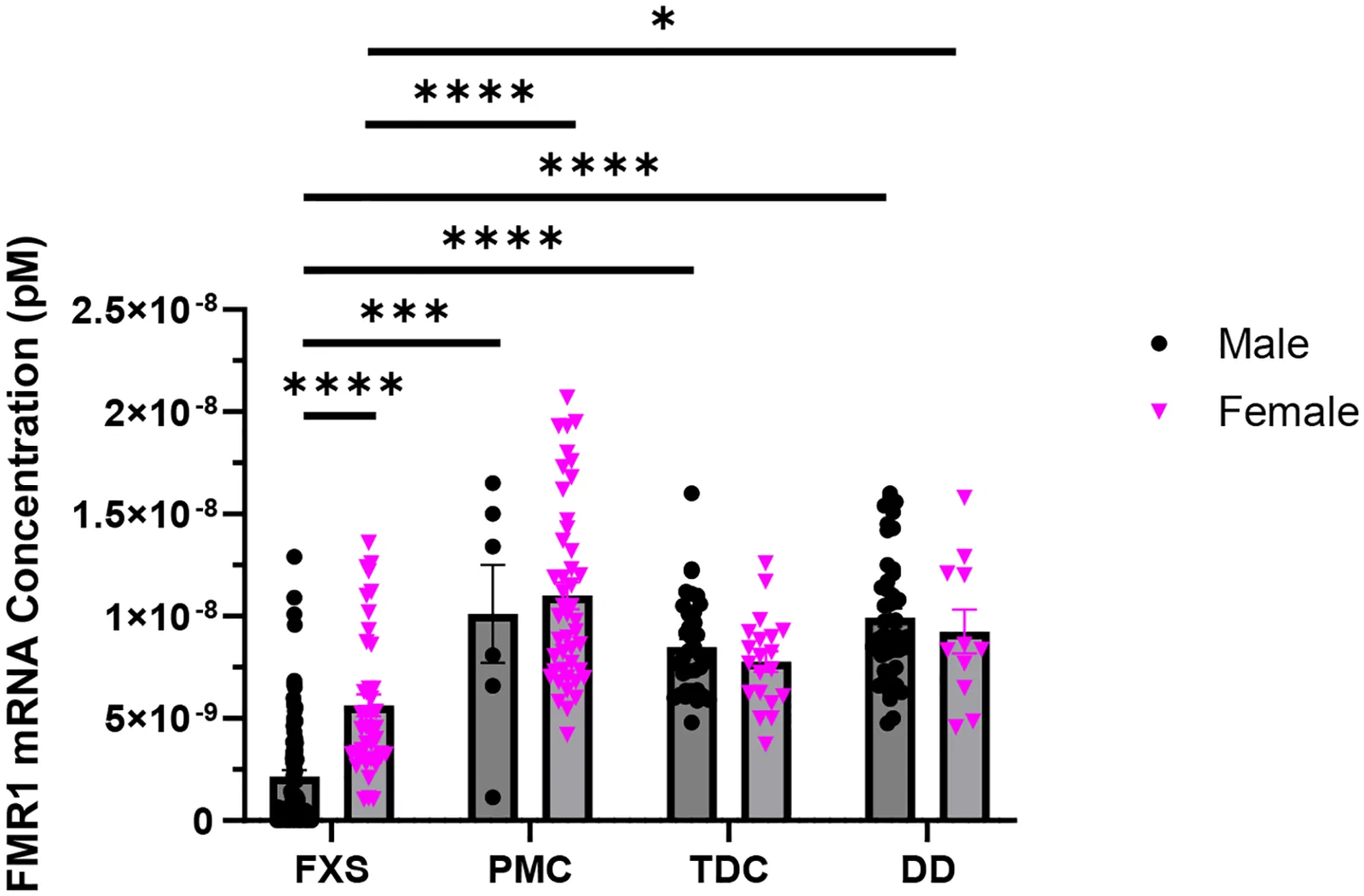
*FMR1* mRNA levels across diagnostic groups and sex. Significant main effect of diagnosis but not sex and significant interaction between diagnosis and sex for *FMR1* mRNA levels in peripheral blood (2-way ANOVA, p(interaction)=0.001, p(diagnosis)<0.0001, p(sex)=0.17). *FMR1* mRNA levels in FXS males are significantly lower than males in all other groups (Tukey’s post hoc tests p<0.001), whereas mRNA levels in females with FXS are significantly lower compared with female PMCs and female DD but not compared with female TDCs. Within-group sex differences were only detected in FXS (p<0.0001). N: male FXS=90; female FXS=41; male PMC=7; female PMC=45; male TDC=36; female TDC=20; male DD=47; female DD=11). Error bars represent SEM. *FMR1*: fragile X messenger ribonucleoprotein 1. PMC: premutation carriers. TDC: typically developing controls. DD: developmental disability control. SEM: Standard Error of the Mean. **** p < 0.0001. *** p <0.001. * p < 0.05.

**Figure 2.**
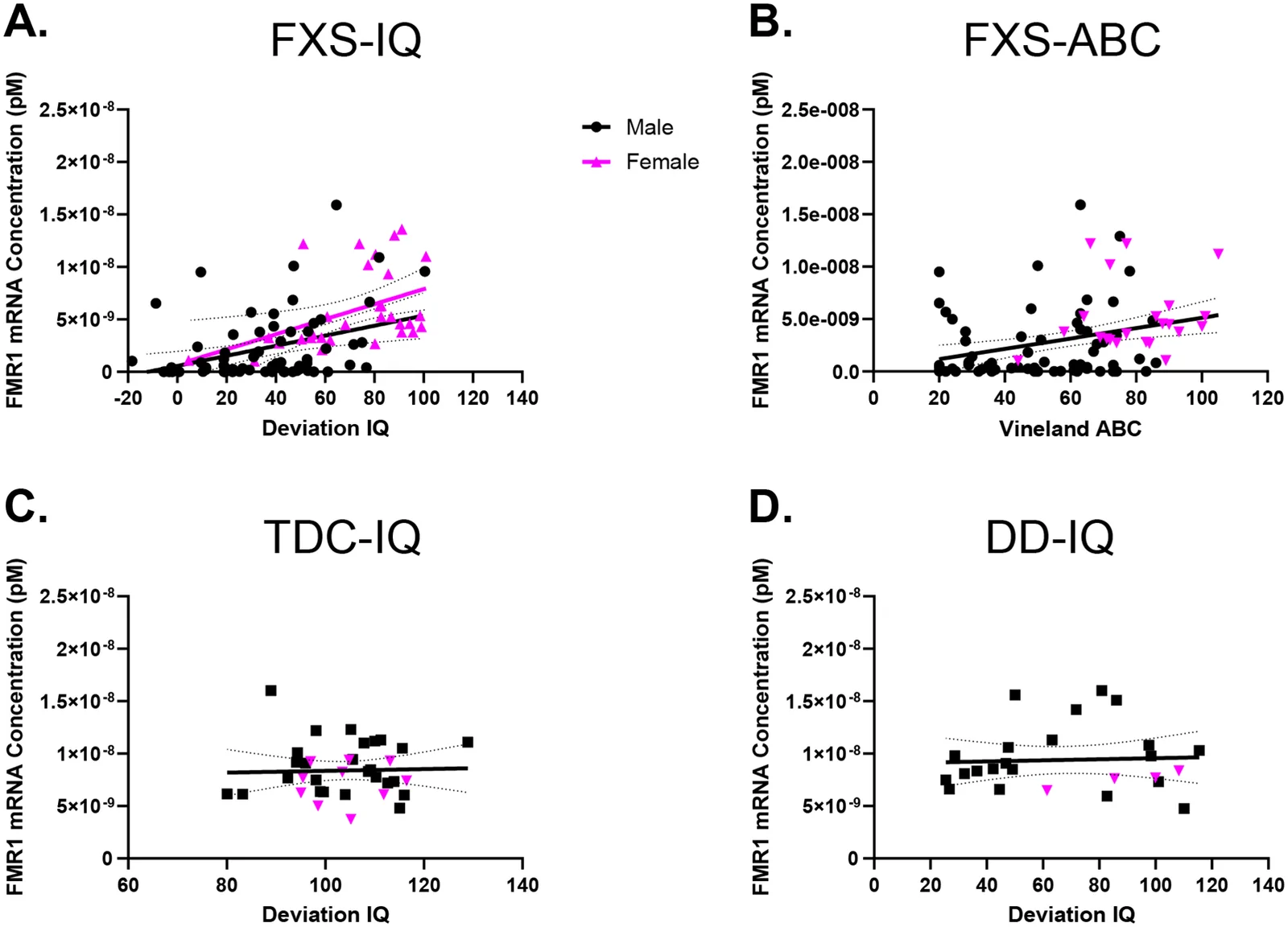
*FMR1* mRNA levels are positively correlated with IQ and adaptive behavior in individuals with FXS. **(A)** Positive correlation of *FMR1* mRNA levels with deviation IQ in male (Pearson’s correlation: n = 64, r = 0.36, R^2^ = 0.13, * p = 0.0038) and female (Pearson’s correlation: n = 31, r = 0.45, R^2^ = 0.20, * p = 0.0119) individuals with FXS, as well as combined (Pearson’s correlation: n=95, r = 0.53, R^2^ = 0.29, p < 0.0001). **(B)** Positive correlation of *FMR1* mRNA with Adaptive Behavior Composite (ABC) in individuals with FXS (Pearson’s correlation: n = 90, r = 0.32, R^2^ = 0.10, * p = 0.0022; no significant correlations when separated by sex). **(C, D)** No significant correlations of *FMR1* mRNA with deviation IQ in TDC (C, Pearson’s correlation: n = 36, r = 0.034, R^2^ = 0.001, p = 0.84) or DD (D, Pearson’s correlation: n = 25, r = 0.05, R^2^ = 0.002, p = 0.81) groups. *Black circles:* male, *pink triangles:* female. *pM*, picomolar

**Figure 3.**
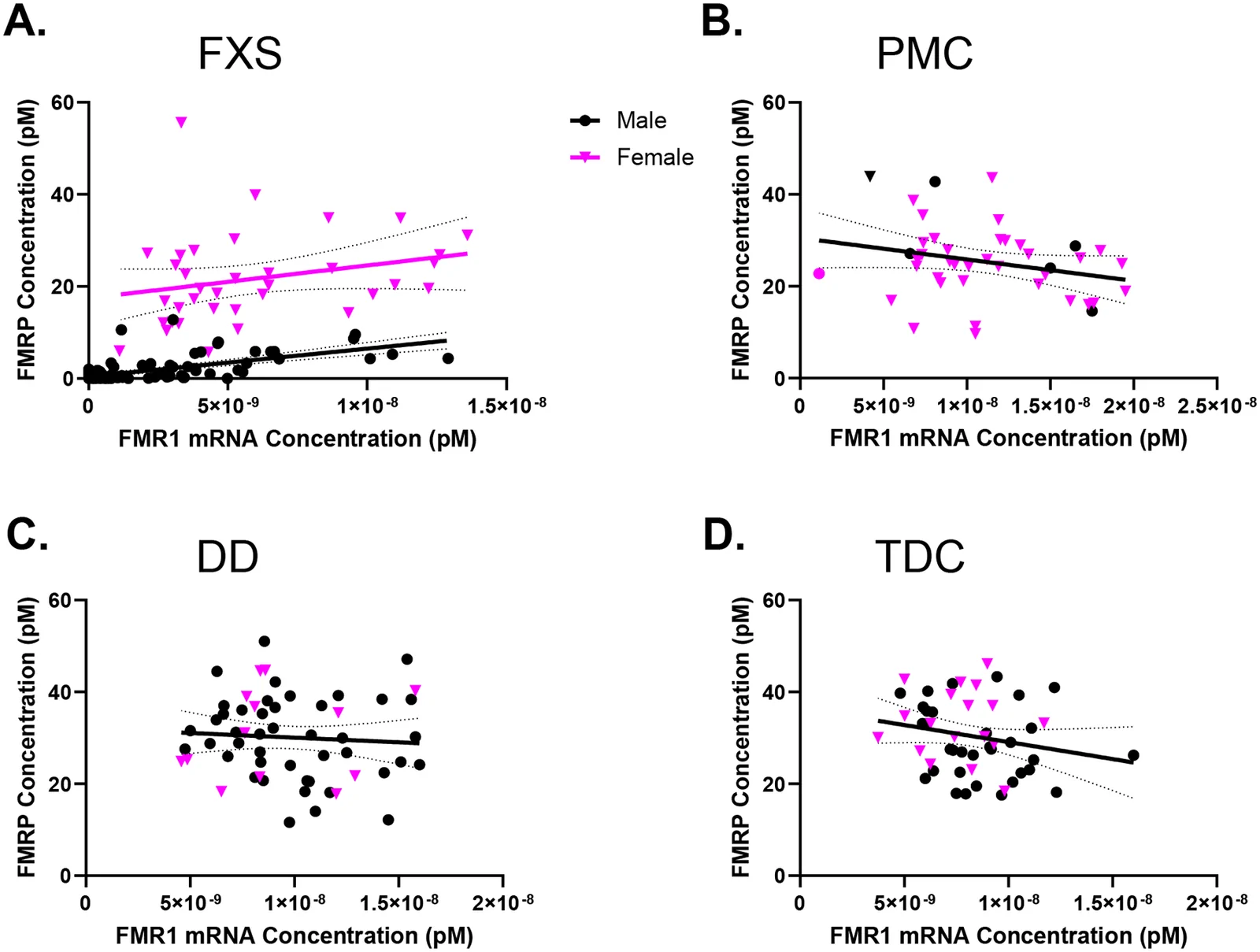
*FMR1* mRNA levels are positively correlated with FMRP in peripheral blood from individuals with FXS. **(A)** Positive correlation of *FMR1* mRNA levels with FMRP in male (Pearson’s correlation: n = 87, r = 0.63, R^2^ = 0.40, p < 0.0001) but not female (Pearson’s correlation: n = 37, r = 0.25, R^2^ = 0.06, p = 0.13) individuals with FXS. **(B-D)** No significant correlations of *FMR1* mRNA with FMRP in PMC (B, Pearson’s correlation: n = 43, r = −0.26, R^2^ = 0.07, p = 0.09), DD (C, Pearson’s correlation: n = 57, r = −0.07, R^2^ = 0.005, p = 0.6) or in TDC (D, Pearson’s correlation: n = 51, r = −0.22, R^2^ = 0.05, p = 0.13) groups. Black circles: male, pink triangles: female. pM, picomolar

**Figure 4.**
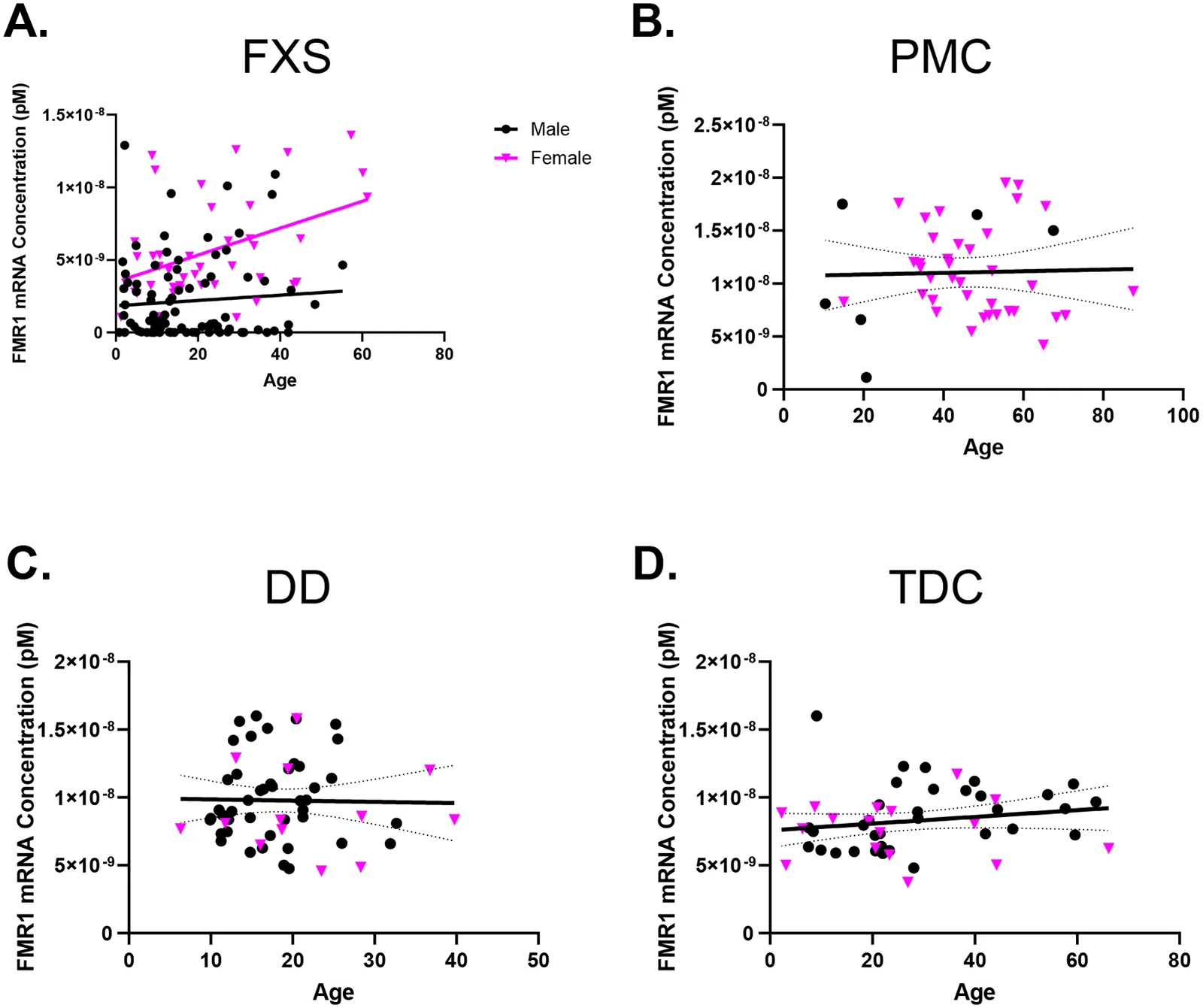
*FMR1* mRNA levels are positively correlated with age in peripheral blood from females with FXS. **(A)** Positive correlation of *FMR1* mRNA levels with age in female (Pearson’s correlation: n = 41, r = 0.42, R^2^ = 0.18, p = 0.006) but not male (Pearson’s correlation: n = 90, r = 0.08, R^2^ = 0.006, p = 0.46) individuals with FXS. **(B-D)** No significant correlations of *FMR1* mRNA with age in PMC (B, Pearson’s correlation: n = 43, r = 0.028, R^2^ = 0.0008, p = 0.89), DD (C, Pearson’s correlation: n = 57, r = −0.02, R^2^ = 0.0004, p = 0.89) or in TDC (D, Pearson’s correlation: n = 51, r = 0.18, R^2^ = 0.03, p = 0.21) groups. Black circles: male, pink triangles: female. pM, picomolar

**Table 1.** *FMR1* mRNA demographic information. *SD*: Standard Deviation.

	N	Age (years)			FMR1 mRNA Concentration (pM)			
		Average	SD	Range	Average	Median	SD	Range
								
**Male:**	179	20.3	13.2	0.8 – 67.5	5.7588E-09	6E-09	4.73E-09	0.0 to 1.75E-08
Fragile X Syndrome	90	17.7	12.2	0.8 – 55.3	2.1619E-09	8E-10	2.82E-09	0.0 to 1.29E-08
Premutation carrier	7	27.2	20.5	9.7 – 67.5	1.1175E-08	1.3E-08	5.60E-09	1.13E-09 to 1.75E-08
Typically developing control	36	28.9	16.2	1.2 – 63.7	8.4784E-09	8E-09	2.35E-09	4.81E-09 to 1.60E-08
Developmental disability	46	17.6	5.4	9.9 – 32.7	9.9346E-09	9.1E-09	3.04E-09	4.75E-09 to 1.60E-08
								
**Female:**	121	33.1	18.7	1.3 – 87.5	8.3636E-09	7.7E-09	4.26E-09	1.03E-09 to 2.07E-08
Fragile X Syndrome	41	23.4	15.5	1.3 – 61.3	5.642E-09	4.6E-09	3.41E-09	1.03E-09 to 1.36E-08
Premutation carrier	45	47.9	13.8	15 – 87.5	1.0997E-08	1E-08	4.38E-09	4.19E-09 to 2.07E-08
Typically developing control	21	28.5	17.5	2.3 – 66.2	7.7293E-09	7.7E-09	2.13E-09	3.74E-09 to 1.26E-08
Developmental disability	14	21.2	9.1	6.3 – 39.8	8.8217E-09	8.2E-09	3.11E-09	4.57E-09 to 1.58E-08

**Table 2.** FMRP demographic information. *FMRP*: Fragile X Messenger Ribonucleoprotein. SD: Standard Deviation.

	N	Age (years)			FMRP Concentration (pM)			
		Average	SD	Range	Average	Median	SD	Range
								
**Male:**	170	20.5	12.9	0.8 – 67.5	15.2	10.1	15.1	0 to 51.02
Fragile X Syndrome	87	18.2	12.1	0.8 – 55.3	1.7	0.3	2.7	0 to 12.76
Premutation carrier	6	30.2	20.7	10.4 – 67.5	26.7	25.6	8.5	14.61 to 42.77
Typically developing control	33	28.7	15.4	7.5 – 63.7	28.8	27.5	7.7	17.58 to 43.31
Developmental disability	44	17.7	5.5	9.9 – 32.7	29.9	30.1	9.0	11.63 to 51.02
								
**Female:**	105	32.6	18.6	2.3 – 87.5	26.0	25.3	9.7	5.70 to 55.60
Fragile X Syndrome	37	24.1	15.8	4.6 – 61.3	21.7	20.3	9.7	5.70 to 55.60
Premutation carrier	37	47.9	13.7	15 – 87.5	25.1	25.4	7.8	9.69 to 43.85
Typically developing control	18	26.6	17.9	2.3 – 66.2	33.3	33.2	7.3	18.45 to 46.11
Developmental disability	13	21.6	9.2	6.3 – 39.8	30.9	31.1	9.4	17.82 to 44.71

**Table 3.** SB-5 demographic information. *SB-5*: Stanford Binet, Fifth Edition. *SD*: Standard Deviation.

	N	Age (years)			SB-5 Deviation IQ Score		
		Average	SD	Range	Average	SD	Range
							
**Male:**	114	21.3	12.0	4.5 – 59.3	58.4	36.5	−18.35 to 128.90
Fragile X Syndrome	64	19.6	11.0	4.5 – 48.5	36.1	24.4	−18.35 to 100.46
Premutation carrier	3	26.5	16.0	10.5 – 48.5	103.5	1.8	100.90 to 105.00
Typically developing control	26	27.2	14.0	7.5 – 59.3	103.8	11.0	80.09 to 128.90
Developmental disability	21	17.9	6.2	10.0 – 32.7	63.7	29.5	25.47 to 115.47
							
**Female:**	55	27.0	17.0	4.7 – 62.2	84.5	23.9	4.61 to 116.46
Fragile X Syndrome	31	23.3	16.0	4.7 – 61.3	71.5	22.7	4.61 to 100.90
Premutation carrier	10	46.1	13.3	15.0 – 62.2	103.6	8.4	89.53 to 112.82
Typically developing control	10	22.3	10.4	6.4 – 44.3	104.1	7.3	95.02 to 116.46
Developmental disability	4	20.2	12.2	6.3 – 39.8	88.7	17.8	61.39 to 108.25

**Table 4.** Vineland-3 ABC demographic information. *ABC*: Adaptive Behavior Composite. *SD*: Standard Deviation.

	N	Age (years)			Vineland ABC Score		
		Average	SD	Range	Average	SD	Range
							
**Male:**							
Fragile X Syndrome	68	15.5	10.4	0.8 – 42.1	49.6	19.8	20 to 86
							
**Female:**							
Fragile X Syndrome	22	15.3	10.6	1.3 – 43.6	79.7	14.5	44 to 105

## Data Availability

Data are shown in tables and graphs in the manuscript. Raw data are available from the corresponding author upon reasonable request.

## List of Abbreviations

ABIQ: Abbreviated Intelligence Quotient
ANOVA: Analysis of Variance
ASD: Autism Spectrum Disorder
cDNA: Complementary Deoxyribonucleic Acid
CGG: Cytosine-Guanine-Guanine trinucleotide repeat
CCHMC: Cincinnati Children's Hospital Medical Center
DD: Developmental Disability (Control Group)
DNA: Deoxyribonucleic Acid
FDR: False Discovery Rate
FM: Full Mutation
FM-FXS: Full-Mutation Fragile X Syndrome
FMR1: Fragile X Messenger Ribonucleoprotein 1 (gene)
FMRP: Fragile X Messenger Ribonucleoprotein (protein)
FXS: Fragile X Syndrome
HC: Healthy Control
IQ: Intelligence Quotient
pM: Picomolar
PMC: Fragile X Premutation Carrier
PMCs: Fragile X Premutation Carriers
qRT-PCR: Quantitative Real-Time Polymerase Chain Reaction
RNA: Ribonucleic Acid
SB-5: Stanford-Binet Intelligence Scales, Fifth Edition
SD: Standard Deviation
SEM: Standard Error of the Mean
TDC: Typically Developing Control
TDCs: Typically Developing Controls
Vineland-3: Vineland Adaptive Behavior Scales, Third Edition

## References

[1] Darnell J.C., , FMRP stalls ribosomal translocation on mRNAs linked to synaptic function and autism. Cell, 2011. 146(2): p. 247–61. 10.1016/j.cell.2011.06.013 PMC3232425 21784246

[2] Penagarikano O., Mulle J.G., and Warren S.T., The pathophysiology of fragile x syndrome. Annu Rev Genomics Hum Genet, 2007. 8: p. 109–29. 10.1146/annurev.genom.8.080706.092249 17477822

[3] Kirchgessner C.U., Warren S.T., and Willard H.F., X inactivation of the FMR1 fragile X mental retardation gene. J Med Genet, 1995. 32(12): p. 925–9. 10.1136/jmg.32.12.925 PMC1051769 8825916

[4] Boggs A.E., , Optimization, validation and initial clinical implications of a Luminex-based immunoassay for the quantification of Fragile X Protein from dried blood spots. Sci Rep, 2022. 12(1): p. 5617. 10.1038/s41598-022-09633-8 PMC8980090 35379866

[5] Straub D., , A sensitive and reproducible qRT-PCR assay detects physiological relevant trace levels of FMR1 mRNA in individuals with Fragile X syndrome. Sci Rep, 2023. 13(1): p. 3808. 10.1038/s41598-023-29786-4 PMC9992378 36882476

[6] Budimirovic D.B., , A Genotype-Phenotype Study of High-Resolution FMR1 Nucleic Acid and Protein Analyses in Fragile X Patients with Neurobehavioral Assessments. Brain Sci, 2020. 10(10). 10.3390/brainsci10100694 PMC7601415 33008014

[7] Taylor S.C., Laperriere G., and Germain H., Droplet Digital PCR versus qPCR for gene expression analysis with low abundant targets: from variable nonsense to publication quality data. Sci Rep, 2017. 7(1): p. 2409. 10.1038/s41598-017-02217-x PMC5445070 28546538

[8] Tassone F., , Elevated FMR1 mRNA in premutation carriers is due to increased transcription. Rna, 2007. 13(4): p. 555–62. 10.1261/rna.280807 PMC1831862 17283214

[9] Tassone F., , Elevated levels of FMR1 mRNA in carrier males: a new mechanism of involvement in the fragile-X syndrome. Am J Hum Genet, 2000. 66(1): p. 6–15. 10.1086/302720 PMC1288349 10631132

[10] Schmitt L.M., , A near normal distribution of IQ in Fragile X syndrome. Sci Rep, 2024. 14(1): p. 23058. 10.1038/s41598-024-73626-y PMC11452685 39367109

[11] McKinney W.S., , Beyond the Fragile X protein: neighborhood characteristics explain individual differences in IQ and adaptive behaviors of Fragile X syndrome. Front Psychiatry, 2025. 16: p. 1636987. 10.3389/fpsyt.2025.1636987 PMC12488564 41048907

[12] Baker E.K., , FMR1 mRNA from full mutation alleles is associated with ABC-C(FX) scores in males with fragile X syndrome. Sci Rep, 2020. 10(1): p. 11701. 10.1038/s41598-020-68465-6 PMC7367290 32678152

[13] Kim K., , Association between IQ and FMR1 protein (FMRP) across the spectrum of CGG repeat expansions. PLoS One, 2019. 14(12): p. e0226811. 10.1371/journal.pone.0226811 PMC6938341 31891607

[14] Sansone S.M., , Improving IQ measurement in intellectual disabilities using true deviation from population norms. J Neurodev Disord, 2014. 6(1): p. 16. 10.1186/1866-1955-6-16 PMC4613563 26491488

[15] Sparrow S.S. and Cicchetti D.V., The Vineland Adaptive Behavior Scales, in Major psychological assessment instruments, Vol. 2. 1989, Allyn & Bacon: Needham Heights, MA, US. p. 199–231.

[16] Verdura E., , Heterogeneity in Fragile X syndrome Highlights the Need for Precision Medicine-Based Treatments. Front Psychiatry, 2021. 12: p. 722378. 10.3389/fpsyt.2021.722378 PMC8514715 34658958

[17] Baker E.K., , Tissue mosaicism, FMR1 expression and intellectual functioning in males with fragile X syndrome. Am J Med Genet A, 2023. 191(2): p. 357–369. 10.1002/ajmg.a.63027 PMC10952635 36349505

[18] Boggs A.E., , Optimization, validation and initial clinical implications of a Luminex-based immunoassay for the quantification of Fragile X Protein from dried blood spots. Scientific Reports, 2022. 12(1): p. 5617. 10.1038/s41598-022-09633-8 PMC8980090 35379866

[19] Roth M., , FMRP Levels in Human Peripheral Blood Leukocytes Correlates with Intellectual Disability. Diagnostics (Basel), 2021. 11(10). 10.3390/diagnostics11101780 PMC8534530 34679478

[20] Yang X., , Oxford Nanopore third generation sequencing for analysis of FMR1 5’UTR CGG repeat expansions. Anal Biochem, 2025. 706: p. 115931. 10.1016/j.ab.2025.115931 40653294

